# Understanding Health Information Behaviors of Migrant Domestic Workers during the COVID-19 Pandemic

**DOI:** 10.3390/ijerph191912549

**Published:** 2022-10-01

**Authors:** Jeffry Oktavianus, Yanqing Sun, Fangcao Lu

**Affiliations:** 1 The Centre for Communication Research, City University of Hong Kong, Hong Kong 999077, China; 2 School of Journalism and Communication, Hunan University, Changsha 410082, China; 3 Department of Family Medicine and Primary Care, The University of Hong Kong, Hong Kong 999077, China

**Keywords:** migrant domestic workers, health literacy, health crisis, misinformation, health information

## Abstract

Migrant domestic workers (MDWs) in Hong Kong remain vulnerable during the COVID-19 pandemic. Obtaining accurate information is essential for MDWs as it helps them understand their predicament and protect themselves. Therefore, this study delves into the MDWs’ health literacy by scrutinizing how they acquire, verify, and respond to pandemic-related information. Semi-structured interviews were conducted with 32 Indonesian MDWs, recruited through purposive and snowball sampling. The data were examined using a constant comparative approach in grounded theory. The findings reveal that the participants engaged in information seeking and scanning to obtain health crisis information, mainly through their friends, family members, and community organizations. The participants also verified the information using their judgment or by consulting other actors, such as local organizations and media outlets. The messages they obtained informed the means to protect themselves, which motivated them to adopt preventive measures. However, some also engaged in maladaptive coping, such as taking ineffective preventive actions. The participants also disseminated health crisis information throughout their social circle. This study concluded that MDWs performed four health information behaviors during the pandemic, namely information acquisition, authentication, sharing, and adoption of preventive measures. However, their information practices may change at different stages of the pandemic.

## 1. Introduction

The coronavirus disease (COVID-19) has affected every corner of society, including disadvantaged groups, such as migrant domestic workers (MDWs), who move abroad to obtain household work [1]. Their vulnerability during the pandemic has been exacerbated by insufficient credible information in their native language regarding the virus and protective measures within the migrant workers’ network [2]. Furthermore, many workers rely on news disseminated on digital platforms [3]. Unfortunately, misinformation about COVID-19 has become rampant on social media within MDW communities [2]. On top of this, many MDWs have inadequate health literacy or the ability to acquire, assess, and understand health information [4], which is a crucial skill for improving their situation during the pandemic [5,6]. Exposure to misinformation and lack of health literacy are detrimental to prevention efforts as they may prevent MDWs from recognizing their susceptibility to the virus and the severity of the health crisis [2]. They also may discourage MDWs from complying with precautionary measures and lead them to engage in poor self-health management behaviors as well as ineffective preventive measures [1,7,8].

Given the importance of health messages and the impacts of the pandemic on MDWs, understanding the health information behaviors of the workers during the pandemic is crucial to improving health communication efforts and preventing disease transmission in the community. However, little research has been conducted on how people process and respond to information amid the COVID-19 pandemic, particularly among disenfranchised communities, such as MDWs, a subpopulation known to be ‘information poor’ [4].

Considering the aforementioned research voids, this study aims to delve into the health literacy of MDWs. In particular, this investigation unravels how MDWs acquire, process, and respond to health information amid the COVID-19 pandemic. This study adopted a qualitative method as it enables researchers to gain meaningful insights into a social phenomenon [9] and comprehend the process of that phenomenon [10], which is the purpose of this study. In particular, interviews were employed to collect the qualitative data because they allowed researchers to examine individuals’ experiences to obtain a deeper understanding of their perspectives [11]. Specifically, in-depth interviews with 32 Indonesian MDWs in Hong Kong were conducted. Hong Kong is a suitable research context to study issues surrounding MDWs as the city is a popular destination for MDWs and home to around 400,000 foreign domestic workers [7], mainly from the Philippines and Indonesia [12]. This investigation contributed to the health communication literature by scrutinizing the health literacy of disenfranchised communities, particularly that of MDWs, through their health information behaviors which remain underexamined. The findings also offer insights to help design future health campaigns that target MDWs.

## 2. Literature Review and Research Questions

### 2.1. Information Acquisition in Times of Health Crises

Crises, including the COVID-19 pandemic, engender uncertainty and ambiguity; thus, individuals, including MDWs, need to acquire information related to such crises to address ambiguity and cope with stress [13]. Information acquisition can be conducted through different practices that Niederdeppe et al. [14] categorized as information seeking and scanning. Information seeking refers to ‘the purposive acquisition of information from selected information carriers’ [15]. This practice involves people’s active efforts to obtain specific information from various sources. For example, Filipino workers in Hong Kong acquire health information from multiple channels, including health professionals (e.g., doctors, nurses), non-professionals (e.g., family members, fellow workers, and employers), community organizations (e.g., migrant unions and faith-based organizations), media outlets, and the internet [16].

On the other hand, information scanning is defined as ‘information acquisition that occurs within routine patterns of exposure to mediated and interpersonal sources that can be recalled with a minimal prompt’ [14]. Unlike information seekers who actively search for and acquire specific news, information scanners stumble upon certain information during general media consumption [17]. In the context of MDWs in Hong Kong, they often are exposed incidentally to health information when they access social media and community media, such as the radio and newspapers [16].

While information can be gathered in many different ways, information acquisition behaviors of MDWs during the pandemic remain underexamined. Thus, this study proposes the following research question:

**RQ1:**  *How did MDWs gather information during the COVID-19 pandemic?*

### 2.2. Information Processing: Internal and External Authentication

Apart from obtaining crisis-related information, individuals also assess the relevance and accuracy of the messages they have obtained [17]. This step is crucial as it allows individuals to understand the ongoing situation better and assists in decision-making response to crises [18]. Individuals can evaluate the relevance and veracity of information in various ways. Tandoc et al. [19] developed a framework known as the audiences’ acts of authentication (3As) model to understand how people verify information. This model comprises internal and external authentication.

People perform internal verification using their judgment to evaluate the credibility of their sources and other message characteristics. When individuals are unsatisfied with this internal process, they consult external resources to verify the news, such as comparing the information with media content or discussing it with others [19]. In the context of MDWs, past studies have indicated that workers in Hong Kong commonly talked to their friends, family members, or community organizations to validate the information they acquired [1]. However, little is known about how MDWs have authenticated information during the pandemic. Thus, the following question is put forth:

**RQ2:**  *How did MDWs evaluate health information during the COVID-19 pandemic?*

### 2.3. Responding to Health Information

Health crisis information that people obtain helps them decide which actions to take in response to a crisis [18]. During the COVID-19 pandemic, the immediate solution for the public, including MDWs, was to take preventive measures to protect themselves and cope with the health crisis [20]. Migrant workers in Hong Kong adopted numerous adaptive and preventive steps to avoid contracting the virus, such as wearing masks, washing hands or using hand sanitizers, staying home, and refraining from attending social gatherings or going to places with fresh COVID-19 cases [1].

However, protection motivation theory [21] posits that aside from adaptive responses, individuals also may engage in maladaptive coping strategies, which became evident during the pandemic [22]. For instance, Oktavianus and Lin [1] discovered that some MDWs in Hong Kong took ineffective measures to protect themselves after receiving misinformation. One worker in their study turned to a vegetarian diet after she was misinformed that eating meat could make people catch COVID-19. These maladaptive coping efforts also take other various forms, including religious faith (i.e., reliance on spiritual belief or faith in God to cope with the impact of the crisis), avoidance (i.e., evading or denying the crisis or its impacts), fatalism (i.e., concluding that nothing can be done to improve the situation), wishful thinking (i.e., relying on unrealistic solutions to cope with the problem, such as miracles), and hopelessness [23]. Considering that MDWs may adopt both adaptive and maladaptive coping strategies, this study seeks to answer the following research question:

**RQ3:**  *How did MDWs respond to health information during the COVID-19 pandemic?*

Aside from implementing solutions, another action that individuals perform during a health crisis is disseminating health information [18]. People frequently distribute health information that they have learned through several communication channels. Regarding MDWs in Hong Kong, they commonly circulate health information and official news throughout their community via messaging applications, such as WeChat [2].

Individuals want to disseminate information for various reasons. Goh et al. [24] identified maintenance of social cohesion in the community as a factor that motivates news sharing. Lu et al. [25] also found that people shared health crisis information during the COVID-19 pandemic to increase awareness and promote preventive actions. However, MDWs’ motivations for sharing news during the pandemic remain understudied. Thus, this study asks the following research questions:

**RQ4:**  *How and why did MDWs share health information during the COVID-19 pandemic?*

## 3. Method

### 3.1. Sampling Procedure and Participants

The data analyzed in this study were derived from in-depth interviews with 32 Indonesian MDWs in Hong Kong in February and March 2020. During this period, COVID-19 had begun to emerge in the city. Hong Kong reported its first COVID-19 cases in late January, and the total confirmed cases reached 714 by the end of March 2020 [26,27]. This study also focused on the experience of Indonesian workers because, unlike their Filipino counterparts, many Indonesian workers cannot speak English fluently [28]. Hence, their access to information in the host society often is limited.

The participants were recruited through purposive and snowball sampling. Purposive sampling was conducted by setting several inclusion criteria for the participants that corresponded to this study’s purpose. First, the participants had to be Indonesian female domestic workers in Hong Kong, as almost all MDWs in Hong Kong are female [29]. Moreover, the workers were employed in Hong Kong during the pandemic. The researchers gained access to the participants through the help of a migrant organization. The lead author, who also was the interviewer, volunteered at the migrant organization as an English teacher and, with the consent of the organization, invited nine students who fulfilled the criteria to participate. These nine participants had known the lead author for at least three months. Apart from that, four participants, who had no prior contact with the researchers, were approached directly in public areas, such as wet markets, apartment lobbies, and parks. Snowball sampling also was employed. Participants and the organizations recommended their friends or the organizations’ members for participation in the study. All invited participants accepted the invitation. Data saturation was achieved after interviews with 20 participants, but 12 more interviews were conducted to confirm the saturation of the data, bringing the final total to 32 participants.

The participants were Indonesian females aged 27 to 50 with an average age of 38. Around 25 participants had attended or completed high school. They had worked in Hong Kong for 6.5 years on average, ranging from three months to 19 years, with an average monthly income of HK$4,472. By comparison, the median monthly earnings of Hong Kong employees in 2020 was HK$18,400, around four times more than the average income of the MDWs examined in this study [30].

### 3.2. Procedure

Semi-structured interviews were conducted via phone calls by the lead author, a male native Indonesian speaker and a Ph.D. candidate at the time of the study. The lead author also previously completed qualitative method coursework and had experience conducting interviews. Before the interviews began, all participants formally agreed to participate in the study by signing consent forms, which also described the researcher’s background and the study’s purpose. They also were made aware that their participation was voluntary and anonymous, and that they could withdraw at any time if they felt uncomfortable during the process. However, all participants completed the interviews.

The semi-structured interviews were conducted in Indonesian, the native language of the participants, with 12 questions related to the research questions (Table 1) and probes. The interviews were audio-recorded for transcription and analysis, and the lead author also took field notes during the process. The interviews lasted between 34 min and 110 min. The participants received a HK$150 shopping coupon for their participation.

### 3.3. Data Analysis

The data were analyzed using a constant comparative approach used in grounded theory [31]. This study also employed an iterative strategy [32] that enabled the researchers to generate the codes inductively from the data while being guided by sensitizing concepts from the literature and research questions. The data were coded by the lead author, who started the data analysis by reading and re-reading the transcripts and field notes to get familiarized with the data. The lead author then coded each line of the transcripts guided by the concepts from the existing literature corresponding to the research questions while also identifying emerging codes from the data [32]. Each line was compared with the previous line, and the codes were modified to fit the new data if necessary [33]. The researcher then identified the patterns and classified the codes into themes or conceptual bins corresponding to the research questions. The results were then reviewed and discussed with the second author, and the codebook was refined accordingly. Following that, the report was produced from these themes with examples from the data. The quotes were translated from Indonesian into English by a research assistant who was a native Indonesian speaker and fluent in English. One of the authors reviewed the translations further. Participants’ identities were replaced with name codes to maintain their anonimity. The analytical process was assisted by NVivo 12 (QSR International, Melbourne, Australia), a software used for qualitative data analysis and management.

This study also followed the methodological rigor criteria defined by Lincoln and Guba [34], which included credibility, transferability, dependability, and confirmability. Credibility was obtained as all interviews were conducted by the same interviewer, who was experienced at collecting qualitative data through interviews. Moreover, this study provided a detailed procedure to ensure transferability. Furthermore, dependability was achieved by providing direct quotes in each code. Finally, the second author reviewed the results of the coding of the first author to ensure confirmability.

## 4. Results

**Figure 1:**
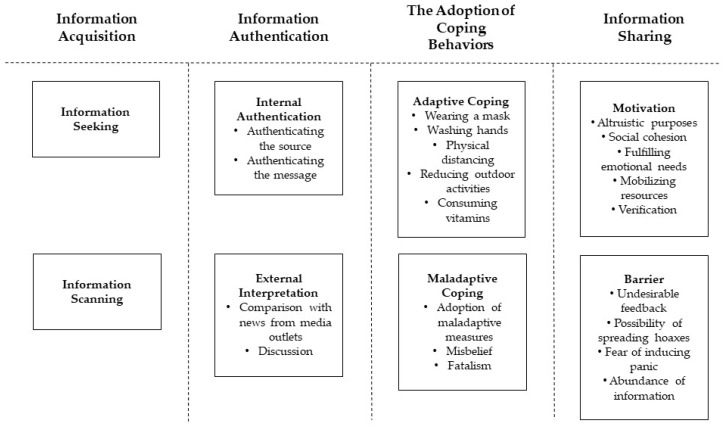
Summary of the participants’ health information behaviors during the COVID-19 pandemic.

The analysis of the interview data uncovered four health information behaviors performed by the workers during the pandemic–information acquisition, information authentication, the adoption of coping behaviors, and information sharing. Figure 1 summarizes these behaviors, and the following sections elaborate on the results.

### 4.1. Information Acquisition: Information Seeking and Scanning

The analysis of the interview data from 32 participants revealed that the workers sought more information about the health crisis after their first encounters with news about the COVID-19 pandemic. The information involved the latest updates regarding the outbreak (e.g., the number and location of positive cases, new government policies), health recommendations (e.g., preventive measures), as well as personal news or the condition of their social circle, particularly their family members and friends. The participants gathered information about the pandemic through *information seeking* and *scanning*.

#### 4.1.1. Information Seeking

Acquiring COVID-19-related news was vital for many participants, including P23 (36 years old), who stated, “*Obtaining information is crucial. If there is no information, I don’t know how to approach this pandemic, like what the solution is*”. Thus, the participants searched for information from multiple channels. Most asked their employers, peers, and family members to learn about the pandemic, such as P12 (50 years old), who explained, “*I asked my friend, like ‘Have you read this news [about the pandemic]?’ They then would give me the information. I also asked my employers because they followed the news*”. The participants also relied on Facebook and WhatsApp accounts of formal local organizations, such as migrant unions and the Indonesian Consulate in Hong Kong, including P23 (36 years old): “*I asked the organization (migrant union) for information about COVID-19. They are trustworthy. They will give us very accurate information*”.

Some participants also monitored several media outlets. Some chose online Indonesian news portals and Indonesian community media in Hong Kong to obtain information related to the pandemic, while a few others who understood English accessed local English-language news websites. Furthermore, some participants watched local television, which broadcasted news in Cantonese, the spoken language of Hong Kong. However, as they commonly had limited Cantonese fluency, participants sometimes relied on their employer to translate the news, such as P17 (39 years old), who stated:

“*I found a lot of news on television, but sometimes I do not understand it (the news). So, I asked my grandmother (the elderly whom she took care of), ‘Grandma, what does it say?’ Then, she would explain it to me”.*

Another participant who actively looked for pandemic-related news through media outlets was P22 (31 years old). She recounted her experience:

“*I know [about coronavirus] from my employers. In the beginning, I only heard that there was a new virus in Wuhan. I was not very responsive at that time because I thought it was just another virus and would not spread anywhere…. But then it spread to many countries and I realized how extraordinary the impact was. When it began to spread in Hong Kong, I didn’t know what to do…. I also worried about whether it would spread to Indonesia. I then found the information about COVID-19 on television. I want to know the latest updates, like the places with infected people or how many people have been infected. I want to know how to avoid the virus”.*

Her story also indicates two motivations for seeking information about the pandemic. First, health crises, such as the COVID-19 pandemic, induce uncertainty. Thus, the MDWs in this study sought information to cope with the unstable situation. Moreover, some participants suggested that they were unsure how to protect themselves from the virus. Therefore, they dealt with this ambiguity by searching for health information, including what precautionary steps to take and the impacts of the pandemic.

The perceived threat also drives information-seeking. When the workers learned about the severity of the pandemic, they became aware that the outbreak elicited detrimental effects on them and others. This perceived threat generated negative emotional responses, such as fear and anxiety. Consequently, they were more motivated to search for updates about the outbreak to find protective measures and anticipate future events.

#### 4.1.2. Information Scanning

However, several participants confessed that they did not seek information about the pandemic and instead performed information scanning. For example, P20 (34 years old) said, “*I don’t look for the news. There are already a lot of people who share the news. I just read the news that I find on Facebook groups or on my timeline [on social media]. Usually, people share the news there*”. Even though she did not find the information intentionally, she was still exposed to the news during her daily communication activities, such as interpersonal conversations and regular social media use, which constituted information scanning.

The findings also revealed an interesting pattern of information acquisition as the workers’ information behavior changed over time. In particular, the analysis noted that many participants gradually shifted from information seeking to information scanning after some time. They admitted that they stopped searching for the news once their information needs were fulfilled and turned to information scanning instead. P5 (42 years old), who was interviewed several weeks after the first report of the positive case in Hong Kong, narrated:

“*I don’t really follow the news about the pandemic anymore. I mean, unlike in the beginning when I searched for information, now I just read it if I see it on social media. I have got used to it [the pandemic]. But I still try to follow the news about government policies…. They keep changing”.*

Her comment indicates that this changing information behavior could occur as the workers were able to control the situation and regulate their emotions after learning information about the health crisis. Thus, the information-seeking practice slowly dissipated, and they tended to scan information instead. Furthermore, this changing information pattern can also occur due to a shift in the point of concern. In the beginning, the workers in this study sought COVID-19 news to understand the situation. After acquiring sufficient knowledge about it, they looked for information related to constantly changing pandemic regulations, which induced uncertainty and directly affected them.

### 4.2. Internal and External Authentications

The information that the participants acquired, however, could be unreliable. P22 (31 years old) noted: “*I feel like the news about COVID from the beginning until now is unclear. Some can be real, and some do not. The news from this source can be different from the other sources. So, I often doubt whether the news is real or not*”. Therefore, some participants also verified their acquired information using their sense of judgment (*internal authentication*) and other stakeholders’ help (*external authentication*).

#### 4.2.1. Internal Authentication: Verifying Source and Messages

Upon receiving information, the participants reported that they determined the veracity of the news using their judgment by focusing on two factors: the *source* and the trustworthiness of the *message*.

The participants noted that when they received information from others, they would consider the reliability of the news source. For instance, P13 (33 years old) shared, “*I only believed in the news from the media, or in Hong Kong, only from television and newspapers. If it came from friends, sometimes the news could be exaggerated, so we often doubted the news”.* Her story implies that the trustworthiness of the source might affect the message’s credibility. When the information sender was viewed as unreliable, the workers tended to filter out the information received from these sources.

P3 (35 years old) also offered another instance. She often checked her social media, such as Facebook, to derive information. When she found a news article shared by her friends online, she tried to identify the original source: “*Like I would see the source of the article. If the source was credible, then I would believe in the news. If not, I would just leave it and ignore it”.*

Her story suggests two layers of message sources to be considered in evaluating the source’s credibility–the ‘visible source,’ or the source that disseminates the information, and the ‘original source,’ or the actors responsible for creating the news items [35]. When the participants were first exposed to news, they judged whether the visible source was reliable. However, when the information came from a third party and this visible source was perceived as unreliable, they would assess the trustworthiness of the original source.

Several participants also said that the characteristics of the message might influence how they processed it. For instance, P25 (35 years old) explained several factors she considered when receiving information: “*I see if it’s (the news) important for me and if it makes sense. If it is not important and sensible, I will just ignore it*”. Given that, the story’s relevance, importance, and plausibility might become indicators to filter out information. When messages lacked personal relevance and credibility, participants stopped processing the information.

#### 4.2.2. External Authentication: Media Outlets and Discussion

Many participants also said that they involved other actors in verifying the news, particularly when they were still unsure about the information’s credibility after internal authentication. For example, P25 (35 years old) stated, “*Sometimes when I see a news story, if it does not make sense, I will ask those who have more knowledge, like my friends who know it better than me*”. The analysis also identified two main actors who helped the participants validate the information: *media outlets* and *other individuals*.

Most participants viewed legitimate and well-established media organizations as credible news sources. Thus, these media outlets often were utilized as references when the MDWs would like to verify information. P7 (45 years old), for instance, narrated:

“*Usually, many friends in the organizations said this and that. We needed to check the information first, whether it was a hoax or not…. I usually watch the news on television in Hong Kong to check, so, I knew if it indeed really happened*”.

The participants also reported that when they doubted the accuracy of information, they preferred to discuss the news with other people whom they perceived trustworthy, as noted by P31 (39 years old):

“*Now, video and text on social media can be edited. It’s hard to discern the hoaxes from real news. So, I try to find the truth from those who understand the issue better, like people I trust. I will ask, ‘I hear from others like this, is it correct?’ If they say it’s correct, then okay, it’s right*”.

Therefore, this cross-checking with other individuals can be one way to authenticate acquired news. Interpersonal communication is a practical approach to confirming news, as others also can corroborate information and validate the message.

Furthermore, the participants noted that the verification process could occur in a group setting, particularly because social media enabled them to have group chats and ask others for their opinions about the credibility of the news. P8 (32 years old), who was a member of a migrant union, stated that WhatsApp groups helped her when she was unsure about the accuracy of information:

“*When I saw the news, I usually asked the group of the migrant union [on WhatsApp] if the news was correct or if it was only a hoax. My friends in the organization checked it together. If the news was a hoax, they would say, ‘Don’t share the news’*”.

Some participants also revealed that they read the comment sections of online posts when they found information on social media, including P13 (33 years old):

“*If there are friends who share something on Facebook, the first thing I will see is the comments. I pay attention to the comment…. If it’s a hoax or inaccurate, there must be many people who comment on the post to correct the information”.*

Her statement highlights the importance of other users’ responses in the comment section, as they are used to clarify and verify information. Opinions reflected in social media comments even can be more convincing when many users convey similar statements because people tend to follow a particular opinion when many share like-minded sentiments.

However, it was notable that several workers might not verify the information. For some, exposure to misinformation was not a concern, or they were unaware of the impact of fake news, as P6 (33 years old) noted: “*I just read it (news about COVID-19). I don’t check if it’s a hoax. I’m not afraid [if the news is misinformation] because I don’t focus on that. I mean, I don’t overthink it*”. Several participants also opted out of external authentication when satisfied with internal verification and directly believed in the information they received. The absence of authentication can pose dangers to participants, as discussed in the following section.

### 4.3. Adoptions of Adaptive and Maladaptive Strategies

Upon learning and assessing information related to the pandemic, the participants performed some *adaptive measures* to avoid contracting the virus and cope with the health emergency. However, it was noteworthy that some participants also took *maladaptive steps*.

#### 4.3.1. Adaptive Coping

After learning about the pandemic, the participants took various preventive measures to protect themselves during the crisis. P31 (39 years old) explained, “*The organization (migrant union) gave information, like how to protect ourselves and maintain our stamina…. Now, I eat and have enough rest. If I come back from outside, I wash my hands and feet. I also just stay at home*”. Similarly, P30 (27 years old) shared, “*I learned from my friends that we need to be careful because the virus is dangerous. I feel scared. So, everywhere I go, I wear a mask and take care of the hygiene*”. Some other preventive steps that the participants performed included showering or spraying disinfectant after returning from outside, limiting the number of large gatherings on Sundays, and taking vitamins.

However, some participants took precautionary steps because of external factors. P7 (45 years old) noted, “*My employer was very talkative. When I came back from outside, she said, ‘You should not touch anything. Take a shower first. Wash your hair, clothes, and shoes….’ I just followed her instructions”.* Thus, she performed the preventive steps partly because her employer asked her to do so. P13 (33 years old) also offered another reason: “*I only wear a mask because it’s compulsory, but I don’t like to wear it. It’s hard to breathe. So, when no one is looking, I take it off*”, suggesting compliance to health protocols due to government regulations. Their comments imply that their adoption of preventive measures may not result from the information-gathering process in previous stages, but rather from external drivers, such as their employers, the government, or their peers.

#### 4.3.2. Maladaptive Coping

The analysis also revealed that some participants consciously and unconsciously adopted *maladaptive actions*, such as taking ineffective preventive measures. This situation was particularly prevalent among those who did not verify the news and believed in misinformation shared by their peers and other sources. Consequently, they took alternative measures to prevent themselves from contracting COVID-19. For instance, when asked about how they protected themselves during the pandemic, P1 (42 years old), who rarely performed the verification process, said, “*I drink jamu (a traditional herbal drink) to build my immune system*”, after her friends told her that the drink could protect her from the virus. However, no scientific evidence regarding the effectiveness of this measure in protecting individuals from COVID-19 existed.

Several participants also developed maladaptive thoughts, such as misconceptions about the pandemic, predominantly from exposure to misleading news. For example, P5 (42 years old) believed that the virus was not real after discussing it with her friends and seeing people’s comments on Facebook posts and YouTube videos:

“*Hong Kong people believe it (the virus) 100%, but there is a business purpose behind this. I told my employer, ‘The virus is not a virus. It’s a business trick for a country to strengthen its economy and ruin other countries’ economy.’ So, we don’t need to respond exaggeratedly. It’s not a virus. It’s because the news sensationalizes it. Also, before the pandemic, alcohol, hand sanitizers, and masks did not sell well in the market. So it’s (the pandemic) business-related*”.

Exposure to these misleading narratives could potentially discourage individuals from taking protective steps. It might reduce the perceived severity of the pandemic or even influence individuals in question to deny the threat, as P5 (42 years old) reiterated: “*I’m not afraid (of the virus). I only follow the regulations to wear a mask and keep physical distances, but I already know (that the virus is a business)*”. Her response indicated that she took preventive steps to comply with the regulations in Hong Kong. She might not have adopted them if they were not mandatory, as she had developed false beliefs about the pandemic.

Another maladaptive strategy was fatalism, which entailed accepting and generating a belief that people could do a little to remedy the situation. For instance, P27 (37 years old) expressed: “*It’s about destiny. If it’s your time to die, you will die”.* Developing maladaptive thoughts, such as fatalism, could be dangerous as they might encourage individuals to disengage from preventive efforts, thereby increasing their risk of contracting COVID-19.

### 4.4. Motivations and Barriers to Sharing Health Information

The participants revealed that aside from performing coping strategies, they disseminated health information, particularly to their peers, employers, and family members in Indonesia. They commonly utilized private social media platforms, such as WhatsApp personal chats, to share the news. However, some preferred communicating publicly on open digital channels, including Facebook posts or WhatsApp statuses.

#### 4.4.1. Motivations to Disseminate Health Information

The participants noted different goals in sharing health crisis information. The analysis identified several motivations, including *altruistic purposes*, *social cohesion*, *fulfilling emotional needs*, *mobilizing resources*, and *verification*.

*Altruistic Purposes*. Most participants reported that they shared news stories to increase the awareness of others, especially about the severity of the health emergency. P10 (45 years old) stated that she gave her friends information about the pandemic: “*They need to know that the virus is dangerous. They can die because of it*”. Many also reminded others to protect themselves during the pandemic. P17 (39 years old) recalled, “*If I meet my friend during a day off and she doesn’t wear a mask, I will teach her and then kindly tell her that this is the season of the virus. We need to take care of ourselves*”.

*Social Cohesion.* The participants also shared information to sustain their relationships with others. For instance, many exchanged personal conditions within their close social circles, such as peers, family members, or local groups or organizations. P28 (43 years old), a member of several online groups for migrant workers in Hong Kong, commented, “*We talked in the group [on WhatsApp]. We shared how we were doing. It’s important for bersilaturahmi (maintaining good ties). If we don’t do that, as time goes by, we can lose contact. Especially we don’t often meet during the pandemic”.* Her response reflects the importance of sharing personal news to build relationships with other workers and sustain regular interactions among social circles.

*Fulfilling Emotional Needs.* Many participants shared information, such as personal information and hard news, to provide emotional comfort to others. P6 (33 years old), for instance, admitted that she informed her family about her condition: “*Because they are far from me, so they need to know my condition here. If we don’t let them know, they will feel worried”.* Therefore, she contacted her family almost every day to reassure them that she was okay.

*Mobilizing Resources.* Some participants also were aware that many needed tangible support, such as masks, hand sanitizers, or even financial resources during the health crisis. Thus, they disseminated information to get help from others. P15 (33 years old) recalled a limited supply of protective gear during the early stages of the pandemic. Her residence in Cheung Chau, an outlying island of Hong Kong, made it more challenging for her and her friends to secure protective supplies. She then posted about the situation in Cheung Chau on Facebook: “*I shared it with everyone, including groups of Indonesian migrant workers communities. Many responded to me like, ‘Ma’am, we have several masks for (workers in) Cheung Chau next week.’ So, there are some donations for my friends and me*”.

*Verification.* In the face of threats from rampant hoaxes, sharing news stories allowed the participants to fact-check the news. For instance, many workers who became members of migrant organizations commonly shared the news on WhatsApp groups with expectations that the officials or peers would verify the information. P31 (39 years old), for example, counted on the migrant union to confirm the piece of information she received, as she said, “*We share the news in the group (on WhatsApp), so others can let us know whether the information is right or not”.*

#### 4.4.2. Barriers to Disseminate Health Information

Although most participants frequently disseminated information to their social circles, some refrained from circulating the news due to *undesirable feedback from others*, *the possibility of spreading hoaxes*, *fear of inducing panic*, and *a large amount of existing information*.

*Undesirable Feedback.* Most participants who decided to keep the news about the pandemic for themselves stated that they did not share the information because of the unfavorable responses they received during the information sharing in the past. Among those who experienced this situation was P32 (37 years old): “*When I told my friends, they just said, ‘Halah’ (an expression of disapproval). Sometimes they don’t believe it. So, I’m tired (of sharing the news)*”. Given the lukewarm or undesirable feedback, she kept the information to herself.

*Possibility of Spreading Hoaxes.* As hoaxes frequently contaminated the information, some hesitated to circulate news items further. P27 (37 years old) admitted, “*I rarely share the news because if I say this and that, but it turns out to be a hoax, I will harm other people*”. Her comment indicates that people’s ability to discern fake news from factual accounts affects not only the information evaluation process but also information-sharing behaviors. When people can identify hoaxes, they have more confidence that their message is correct and may be more likely to share it. On the other hand, a lack of such skills and knowledge hinders health information dissemination.

*Fear of Inducing Panic.* Some participants also selectively shared information about the pandemic, particularly when it contained negative news, as they feared generating adverse psychological impacts, such as anxiety and fear, which was the case for P19 (39 years old): “*I felt very anxious and scared during this pandemic. However, I only confided these feelings to my friends. If I shared it with my family, they would panic*”.

*The Abundance of Information.* During the pandemic, vast information streams were emanating from different sources, and some participants stated that they did not need to distribute this news further. For example, P30 (27 years old) said, “*There is a lot of news about the virus already. I feel like my friends already know*”. Therefore, she felt that it was unnecessary to share and repeat the information to her peers.

## 5. Discussion

This study examined the health information behaviors of MDWs amid the COVID-19 pandemic. The findings demonstrate that MDWs underwent several steps in processing health information, including information acquisition, evaluation and authentication, adoption of actions, and information dissemination. This study enriches the literature on health communication and informs policymakers regarding effective health campaign strategies that target marginalized populations, particularly MDWs. This present research can serve as the groundwork for understanding the health literacy of disadvantaged groups, especially how they process health information during infectious disease outbreaks.

### 5.1. Changing Pattern of Information Acquisition

One key finding from this study is the prevalence of health information seeking during the early stages of the pandemic. The interview data suggest two motivations for this information-seeking behavior. First, the workers searched for more news after learning about the impact of the pandemic, suggesting that perceived threat played a role in motivating information seeking. Perceived threat induces negative emotions, such as anxiety, spurring people to take action to eliminate these feelings by seeking information [36]. This finding is consistent with studies by Zhou et al. [37] and Huang and Yang [38], which revealed that perceived threats triggered affective responses that encouraged people to seek more information about the health crisis. However, unlike past literature that primarily focused on fear induced by the perceived threat to the self [37], migrant workers were concerned not only with their own safety but also about their family members at home who lived separately from them. Therefore, they also sought information about Indonesia, as they were anxious about the effects of the pandemic on their families.

Apart from that, the workers also sought information to manage uncertainty that the pandemic triggered, which could occur because unprecedented events, including health crises, may generate pervasive ambiguity. However, individuals cannot define ambiguous situations, and thus, they seek more information [13]. This ambiguity mainly comes from the newness of the virus and the unpredictable impacts of the outbreak. Thus, the workers engaged in information-seeking practice to obtain a clearer picture of the situation and minimize these ambiguities. Past studies also have reported similar findings. For instance, Tang and Zou [39] discovered that residents of Hubei, China, actively sought information mainly during the early stage of the pandemic when the uncertainty level was high.

Although information seeking was prevalent, especially at the beginning of the pandemic, the findings indicate that this behavior slowly dissipated and that more workers performed information scanning instead. One possible explanation is that people were able to manage the ambiguity generated by uncertain circumstances over time. Therefore, the workers stopped looking for information and instead began information scanning. This finding corresponds with the work of Tang and Zou [39], which asserted that uncertainty level, perceived risk, and information needs evolved throughout different phases of the health crisis. Consequently, individuals changed their information practices based on the situation. Therefore, future public health campaigns targeting MDWs during crises should adjust communication strategies throughout the various stages of health emergencies.

### 5.2. Reliance on Informal Networks for Information Acquisition

The findings of this study also shed light on the importance of friends, family, and members of community organizations as information sources in times of health crisis. The reliance on informal networks to obtain pandemic-related news may intensify because many Indonesian workers are not fluent in Cantonese. Simultaneously, the mainstream media, particularly local television, does not provide information in Indonesian [1]. Hence, MDWs in this study turned to Indonesian communication actors who spoke the same language to obtain news, such as fellow workers, Indonesian organizations in Hong Kong, and community media. This finding also confirms previous studies’ assertions that informal networks serve as a vital source of health information for MDWs in times of crisis [1,3]. Nonetheless, MDWs’ limited language skills and lack of news in their native language may affect their health literacy practices by preventing them from acquiring information in the local context. Given this, health promotions for migrant workers are encouraged to provide more information in the mother tongue of the migrant communities and incorporate stakeholders who share the same nationalities into the campaigns to reach MDW communities more effectively.

Despite the benefits of interpersonal networks, relying on them to acquire health information also comes with potential disadvantages. For example, the messages can contain misinformation. The present study’s participants reported receiving different forms of fake news from their peers and family members. Similar findings are also found in previous studies. For instance, Liem et al. [3] discovered that many Indonesian MDWs were exposed to misleading information from unverified sources. Another study by Oktavianus and Lin [1] also found that inaccurate information often contaminated news from fellow workers and family members. Exposure to this health misinformation can elicit detrimental effects on the workers, such as adoption of maladaptive coping strategies. Therefore, finding solutions to health misinformation is essential.

### 5.3. Authenticating Information Internally and Externally

One way to break the chain of misinformation is through information authentication. Many MDWs in this study suggested that they assessed the veracity of the news by using their judgment and the assistance of other actors, such as media outlets, peers, and local organizations. This finding is in line with the work of Tandoc et al. [19], suggesting that people may perform internal and external authentications to validate the information. In a similar vein, Bautista et al. [40] have also suggested that individuals engage in internal and external acts of authentication. People commonly start the process by evaluating their knowledge and skills (internal authentication). When this internal process is insufficient, individuals often turn to other sources to assess the information (external authentication).

The findings also indicate that external verification can be performed at individual and group levels. Some participants chose to discuss the news with their friends or family members individually, while others admitted to relying on local groups (e.g., migrant unions) to evaluate the news they acquired. This collaborative practice constitutes ‘social authentication’ or a group behavior to validate news collectively by sharing information and providing corroboration [41]. This practice not only helps evaluate information accuracy, but also enables the group to maintain cohesion.

However, several workers were not motivated to verify such information. Moreover, they gathered news from untrustworthy online sources, which were prone to misinformation contamination. Some participants also relied on their judgment to verify the news, as noted earlier, but this self-validation requires adequate knowledge and skills to determine the veracity of information [40]. Unfortunately, MDWs are known to be ‘information poor’ due to limited access to credible news [4]. Furthermore, many workers lack information literacy [42]. Hence, they may not have sufficient understanding or knowledge to verify the information by themselves. Consequently, internal authentication can be ineffective, and the workers may be potentially misguided. Therefore, improving their health literacy by equipping them with accessible and reliable health information is crucial to enhancing their skills and capacities to assess information.

### 5.4. Health Misinformation and the Adoption of Maladaptive Actions

The findings also indicate that the workers responded to health crisis information by adopting various coping strategies, including adaptive and maladaptive behaviors. Most workers took adaptive measures by performing the recommended precautions, such as mask-wearing and handwashing. Moreover, consistent with the literature on health [22,36], the participants’ narratives indicated that perceived threat was one factor that drove them to practice protective behaviors. When they learned about the pandemic from the news and sensed that it could negatively impact them as well as their surroundings, they felt the need to take action to protect themselves and others. However, it is also noteworthy that some participants engaged in preventive behaviors due to external drivers, such as their employers’ commands, peer influence, or enacted regulations. This finding is consistent with past studies arguing that people adopt preventive measures and other health behaviors during the COVID-19 pandemic because of not only news consumption, but also other factors, such as social norms and their surroundings [43,44].

This study also discovers that some participants adopted maladaptive coping strategies, including performing ineffective measures as well as developing faulty beliefs and fatalism. One contributing factor to these behaviors was incomplete verification of information. Some participants were exposed to misleading information regarding the pandemic, as they could not discern fake news from real news. Therefore, they were misinformed and misled. Liem et al. [2] noted that inaccurate pandemic-related information remains active on social media of MDWs in Hong Kong and Macao, inducing panic and amplifying stigmatization of infected individuals.

### 5.5. Benefits and Barriers of Information Sharing

Apart from adopting coping strategies, the participants circulated the news to their social circle. The analysis has noted several goals for disseminating COVID-19-related information, including altruistic purposes, maintenance of social cohesion, fulfillment of emotional needs, mobilization of resources, and verification. This finding indicates that news sharing functions not only as informational support (i.e., updating others about the latest situation, verification) but also as instrumental and emotional aids, given that these workers can mobilize resources and comfort others through information dissemination.

Furthermore, one of the recurring themes in the interviews was the role of information sharing in maintaining relationships with other people. In particular, news sharing enabled these workers to continue having regular conversations with others while maintaining established ties during the pandemic. This finding resonates with the work of Goh et al. [24], which discovered that reciprocal news exchange could facilitate social cohesion maintenance, as interactions are a social lubricant that helps sustain relationships in social groups.

Although information sharing can elicit positive consequences, some workers hesitate to disseminate COVID-19-related news for various reasons. One that is particularly concerning is the fear of spreading hoaxes in their social circle. This finding indicates that some MDWs are not confident with their own ability to assess the veracity of the news, and thus, they avoid engaging in information sharing. Previous studies also have found that health literacy has affected the frequency of information sharing during the pandemic. In other words, people with higher health literacy are more likely to disseminate information [45]. Therefore, more effort should be devoted to enhancing the health literacy of MDWs, which may improve their skills in evaluating information, allowing them to disseminate reliable news, which is helpful in informing others about the latest updates and correcting health misinformation.

### 5.6. Limitations, Future Research Directions, and Contributions

The findings of this study contain some limitations. This investigation collected data during the early stages of the pandemic. However, the information behavior can shift over time. Therefore, future studies should conduct longitudinal research to examine how the process changes throughout various stages of a crisis. Furthermore, this study focused only on female Indonesian MDW populations in Hong Kong. Other genders (e.g., male workers), ethnic groups (e.g., Filipina MDWs), and contexts (e.g., Singapore and Malaysia) were not represented in this study. However, they may have different levels of health literacy and health information behaviors. Hence, subsequent investigations may want to scrutinize the response process of other MDW populations or even compare the information practices of different groups.

Despite these limitations, this study enriches the health communication literature by delving into health information behaviors of disenfranchised communities during health emergencies, a topic that remains underexplored. Understanding how MDWs gather, evaluate, respond to, and share health information during the pandemic also offers practical insights that the government and other institutions can use when designing future crisis response strategies that target this community. This study discovers that the information needs and behaviors change at different stages of the health crisis. Hence, public health campaigns should be adjusted through various phases of future crises. Moreover, misinformation became a major concern in MDW communities during the pandemic. Unfortunately, several workers did not verify the information they received and even gathered news from unreliable sources that are prone to misinformation. This situation was exacerbated by the lack of credible information circulating in the MDWs’ networks. Hence, improving digital media and health literacy skills is pivotal in educating these workers on how to discern fake news to combat misinformation. Improved health literacy also can build confidence in these workers’ ability to correct hoaxes they encounter, which can help fight against misinformation. Moreover, it is crucial for official channels, such as the Indonesian Consulate, local health agencies, and non-profit organizations, to supply credible information, as the absence of official information can contribute to the spread of misinformation.

## 6. Conclusions

This study unravels the health information behaviors of MDWs in Hong Kong during the COVID-19 pandemic. The analysis of interview data with 32 Indonesian workers in Hong Kong discovers that the participants derived information regarding the pandemic from various sources, including informal networks and media outlets. Their information acquisition behaviors, however, changed at different stages of the pandemic. At the beginning of the pandemic, they actively and purposefully sought COVID-19-related information, but after some time, they engaged in information scanning instead. They also evaluated the news they acquired via internal and external authentications. However, some workers had limited knowledge and health literacy, which might make their internal authentication ineffective. Apart from this, information in their native language was lacking, which might affect the external authentication process, especially when they would like to compare the information they received with media content. Moreover, the participants responded to the information by adopting adaptive and maladaptive behaviors, aside from disseminating the messages throughout their social circles. Furthermore, the analysis diagnosed problems that may emerge during information processing, such as misinformation, limited information resources in the host society, and a lack of health literacy that might have affected how MDWs cope with the health crisis.

## Figures and Tables

**Table 1:** Interview guide.

**Information Acquisition**	
1.	How did you first get the news about COVID-19? What was your reaction at that time?
2.	How do you get news related to the COVID-19 pandemic?
3.	What information do you try to find or get from these sources?
4.	Why do you choose your information sources? Do you think they are trustworthy?
**Information Authentication**	
5.	Do you usually verify the information you receive? Why?
6.	How do you evaluate the information you obtain?
**Adoption of Coping Behaviors**	
7.	What did you do to protect yourself during the pandemic?
8.	Why did you decide to take that action?
**Information Sharing**	
9.	Do you usually share information about COVID-19? To whom and how?
10.	What information do you usually share?
11.	Why do you want or not want to share news about COVID-19?
12.	Are there any challenges or barriers to sharing information about COVID-19?

## Data Availability

Data sharing is not applicable to this article.
